# Mixed Convection Peristaltic Flow of Third Order Nanofluid with an Induced Magnetic Field

**DOI:** 10.1371/journal.pone.0078770

**Published:** 2013-11-18

**Authors:** Saima Noreen

**Affiliations:** Department of Mathematics, Comsats Institute of Information Technology, Park Road, Chak Shahzad, Islamabad, Pakistan; Gazi University, Turkey

## Abstract

This research is concerned with the peristaltic flow of third order nanofluid in an asymmetric channel. The governing equations of third order nanofluid are modelled in wave frame of reference. Effect of induced magnetic field is considered. Long wavelength and low Reynolds number situation is tackled. Numerical solutions of the governing problem are computed and analyzed. The effects of Brownian motion and thermophoretic diffusion of nano particles are particularly emphasized. Physical quantities such as velocity, pressure rise, temperature, induced magnetic field and concentration distributions are discussed.

## Introduction

Peristaltic motion is now an important research topic due to its immense applications in engineering and physiology. This type of rhythmic contraction is the basis of peristaltic pumps that move fluids through tubes without direct contact with pump components. This is a particular advantage in biological/medical applications where the pumped material need not to contact any surface except the interior of the tube. The word “peristalsis” comes from a Greek word “Peristaltikos”which means clasping and compressing. The peristaltic flow has specific involvement in the transport of urine from kidney to the bladder, chyme movement in gastrointestinal tract, movement of ovum in the female fallopian tubes, blood circulation in the small blood vessels, roller and finger pumps, sanitary fluid transport and many others. Latham [1] and Shapiro et al. [2] reported initial studies for the peristaltic flow of viscous fluid. Since then ample attempts have been made for peristalsis in symmetric flow geometry (seefewrecentstudies [3]–[8]). Recently, physiologists argued that the intra-uterine fluid flow (because of mymometrical contractions) represents peristaltic mechanism and the myometrical contractions may appear in both asymmetric and symmetric channels [9]. Hence some researchers [10]–[15] discussed the peristaltic transport in an asymmetric channel with regard to an application of intra-uterine fluid flow in a nonpregnant uterus.

Heat transfer in cooling processes is quite popular area of industrial research. Conventional methods for increasing cooling rates include the extended surfaces such as fins and enhancing flow rates. These conventional methods have their own limitations such as undesirable increase in the thermal management system's size and increasing pumping power respectively. The thermal conductivity characteristics of ordinary heat transfer fluids like oil, water and ethylene glycol mixture are not adequate to meet today's requirements. The thermal conductivity of these fluids have key role in heat transfer coefficient between the heat transfer medium and heat transfer surface. Hence many techniques have been proposed for improvement in thermal conductivity of ordinary fluids by suspending nano particles in liquids. The term “nano” introduced by Choi [16] describes a liquid suspension containing ultra-fine particles (diameter less than 50 nm). The nanoparticle can be made of metal, metal oxides, carbide, nitride and even immiscible nano scale liquid droplets. Although the literature on flow of viscous nanofluid has grown during the last few years (see [17]–[32] andmanyrefs.therein) but the information regarding peristaltic flow of nano fluids is yet scant. To our information, Akbar and Nadeem [33] studied the peristaltic flow of viscous nanofluid with an endoscope. Influence of partial slip in peristaltic flow of viscous fluid is explained by Akbar et al. [34].

The aim of present study is to venture further in the regime of peristalsis for fluids with nanoparticles. Therefore we examine here the mixed convective peristaltic transport of third order nanofluid in an asymmetric channel. Channel asymmetry is produced by peristaltic waves of different amplitude and phases. Mathematical modelling involves the consideration of induced magnetic field, Brownian motion and thermophorsis effects. Numerical solution of nonlinear problem is obtained using shooting method. Limiting case for viscous nanofluid in symmetric channel is also analyzed. Detailed analysis for the quantities of interest is seen.

## Physical Model

Extra stress tensor 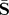 for third order fluid model is given by
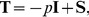
(1)

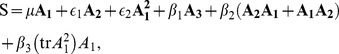
(2)

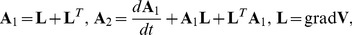
(3)in which 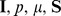 and 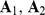 respectively stand for the identity tensor, the pressure, the fluid dynamic viscosity, the extra stress tensor and the first and second Rivlin Ericksin tensors in which the material parameters 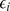; 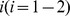 and 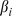; 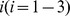 must satisfy
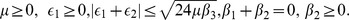
In the absence of displacement current, the Maxwell's equations are
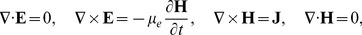
(4)

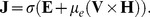
(5)


## Mathematical Formulation

Consider third order nanofluid in an asymmetric channel of width 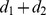. Let 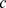 be the speed by which sinusoidal wavetrains propagate along the channel walls. The 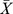 and 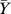-axes in the rectangular coordinates 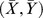 system are taken parallel and transverse to the direction of wave propagation, respectively. A constant magnetic field of strength 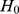 acts in the transverse direction resulting in an induced magnetic field 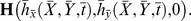 The total magnetic field is 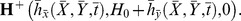 Further the lower wall is maintained at temperature 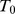 and nano particles concentration 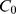 while the temperature and nanoparticles concentration at the upper wall are 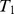 and 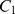 respectively. The wall surfaces satisfy
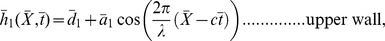


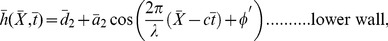
(6)where 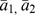 are the wave amplitudes and the phase difference 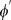 varies in the range 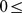 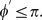 The case 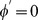 is subjected to the symmetric channel with waves out of phase and the waves are in phase for 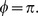 Further 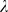 is the wavelength, 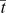 the time and 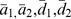 and 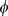 satisfy 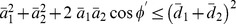. Denoting the velocity components 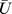 and 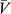 along the 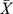 and 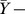
directions in the fixed frame, one can write 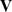 as
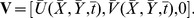
(7)The fundamental equations governing the flow of an incompressible fluid are
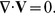
(8)

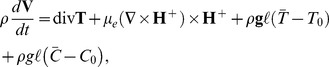
(9)

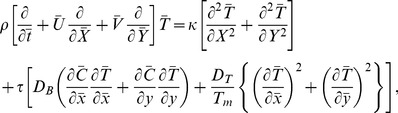
(10)

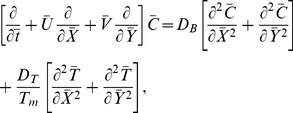
(11)

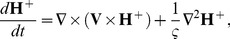
(12)in which 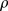 denotes the density of fluid, 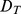 the thermophoretic diffusion coefficient, 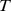 the temperature, 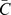 the concentration, 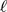 the thermal conductivity, 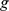 the acceleration due to gravity, 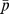 is the pressure, 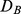 is Brownian diffusion coefficient, 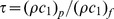 the ratio of the specific heat capacity of the nanoparticle material and heat capacity of the fluid, 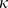 the thermal diffusivity, 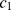 is the volumetric volume expansion coefficient, and 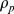 is the density of the particle, 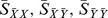 are components of extra stress tensor 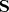 and 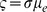 is the magnetic diffusivity.

To facilitate the analysis, we introduce the following transformations between fixed and wave frames
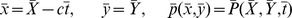


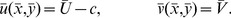
(13)in which (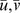) are the velocity components in the wave frame.

Equations (1)–(12) in terms of above transformations give
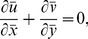
(14)

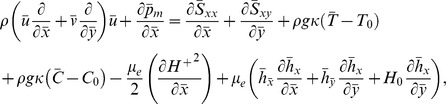


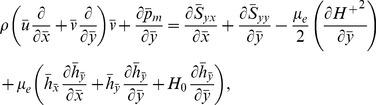
(16)

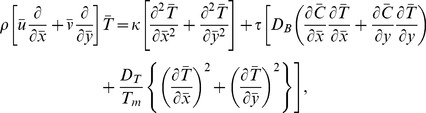
(17)

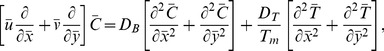
(18)

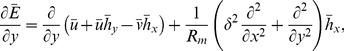
(19)Defining 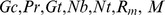 as mass Grashof number, Prandtl number, local temperature Grashof number, Brownian motion parameter, thermophoresis parameter, magnetic Reynold number and Hartman number
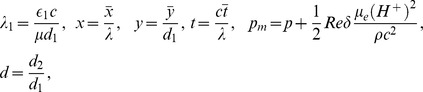


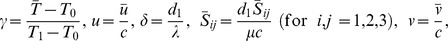


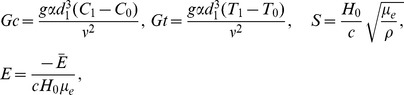


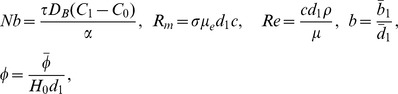


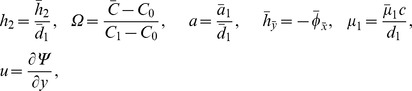


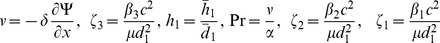


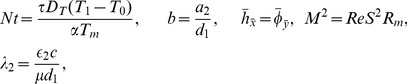
(20)and then employing long wavelength and low Reynolds number approximation, the dimensionless forms of above equations in terms of stream function 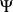 and magnetic force function 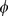 can be expressed as
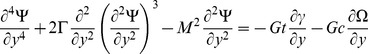
(21)

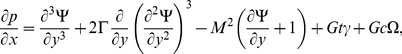
(22)

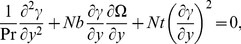
(23)

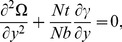
(24)

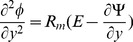
(25)where 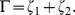


The dimensionless boundary conditions are given by
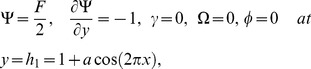


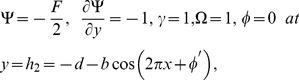
(26)with 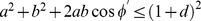. The dimensionless time mean flow rate 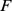 in the wave frame is related to the dimensionless time mean flow rate 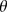 in the laboratory frame by the following expressions
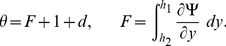
(27)


## Results and Discussion

Our main interest in this section is to examine the velocity (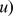, temperature (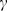), concentration (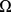), pressure rise per wavelength (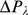), induced magnetic field 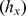 for the influence of local Grashof number (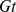), Deborah number (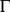), mass Grashof number (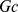), Prandtl number (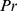), Brownian motion parameter (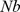), Hartman number (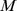), magnetic Reynolds number (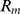) and thermophoresis parameter (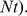


### 4.1. Pumping characteristics

**Figure 1:**
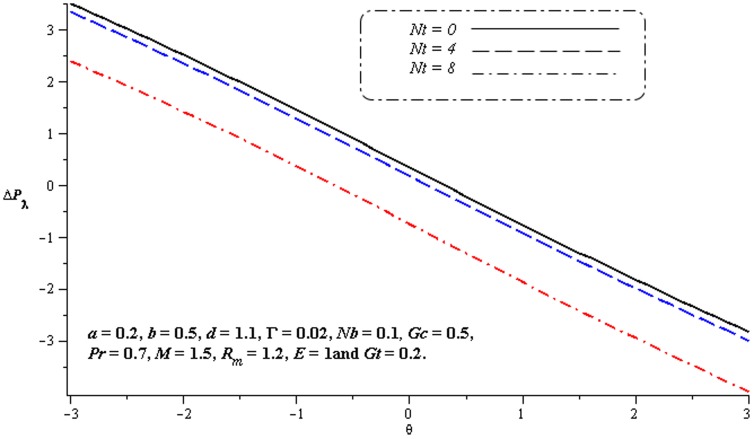
Influence of 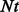 on 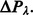

**Figure 2:**
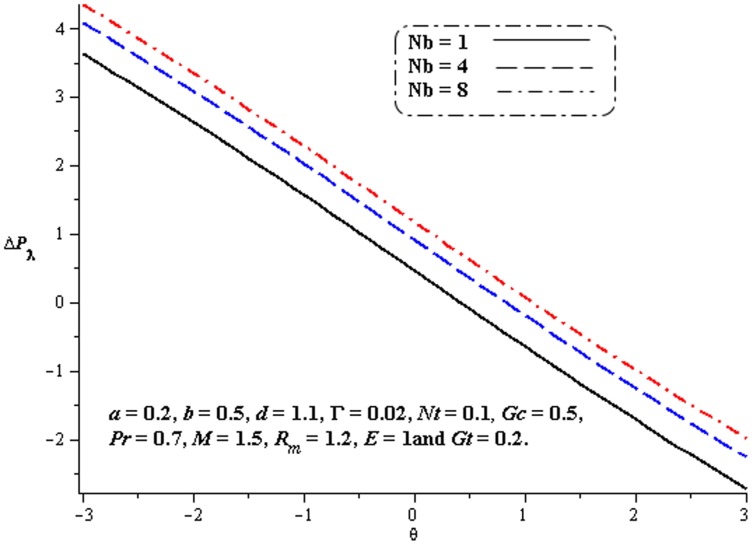
Influence of 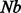 on 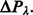

**Figure 3:**
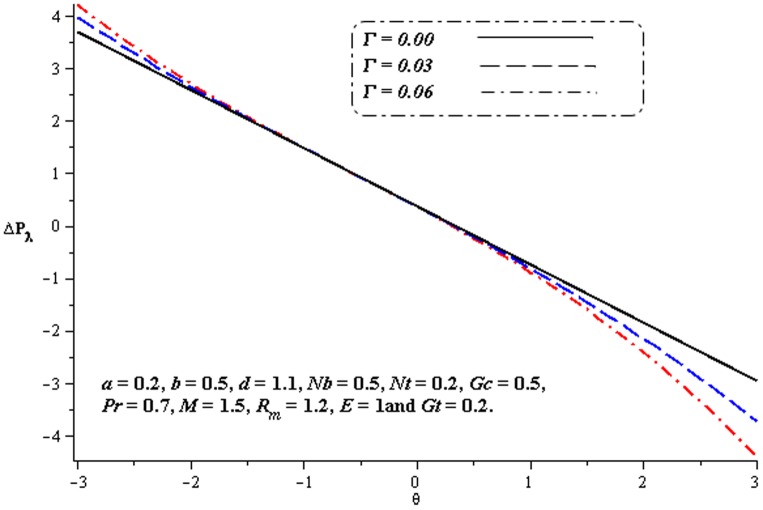
Influence of 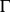 on 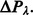

**Figure 4:**
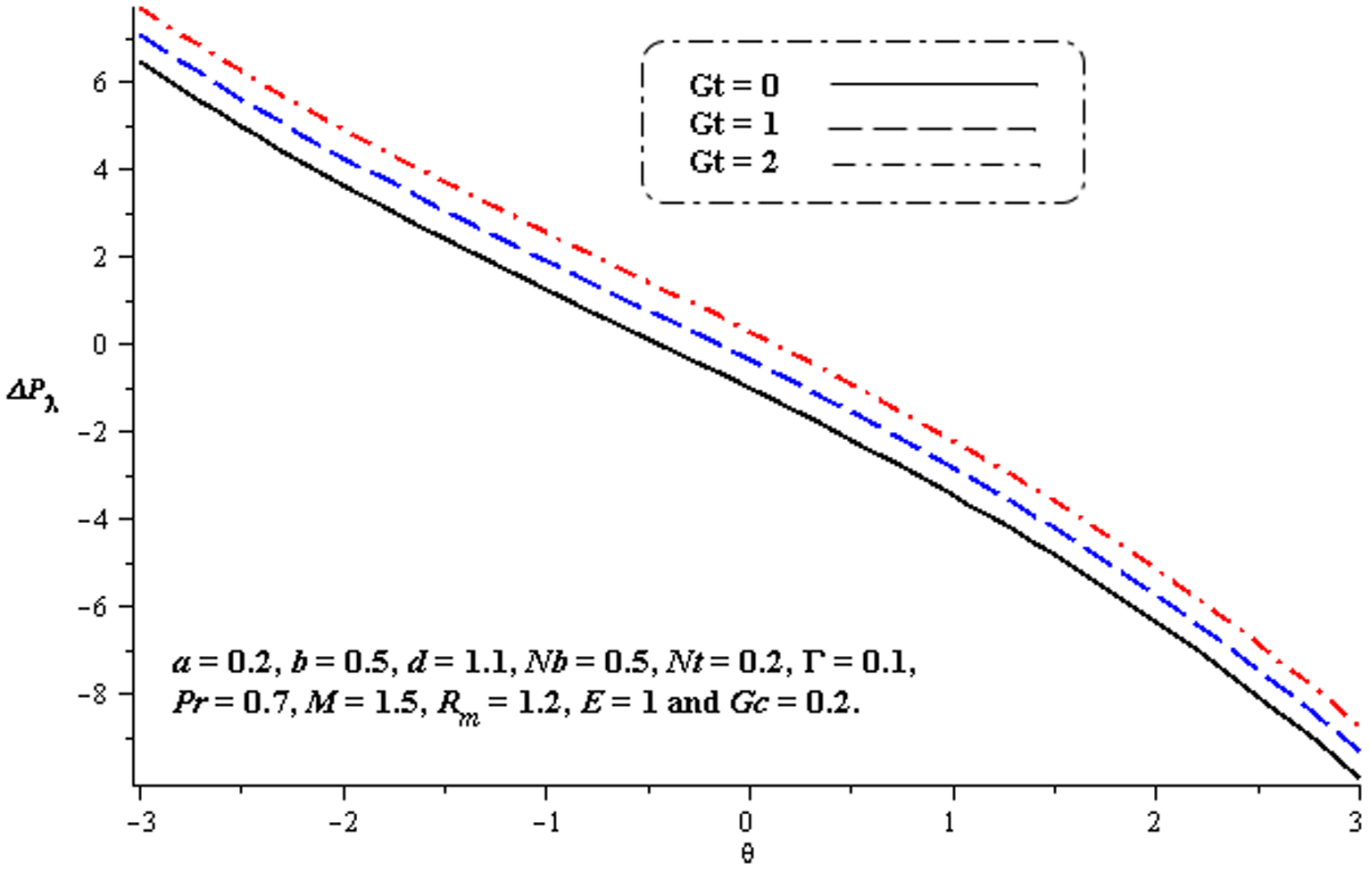
Influence of 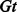 on 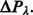

**Figure 5:**
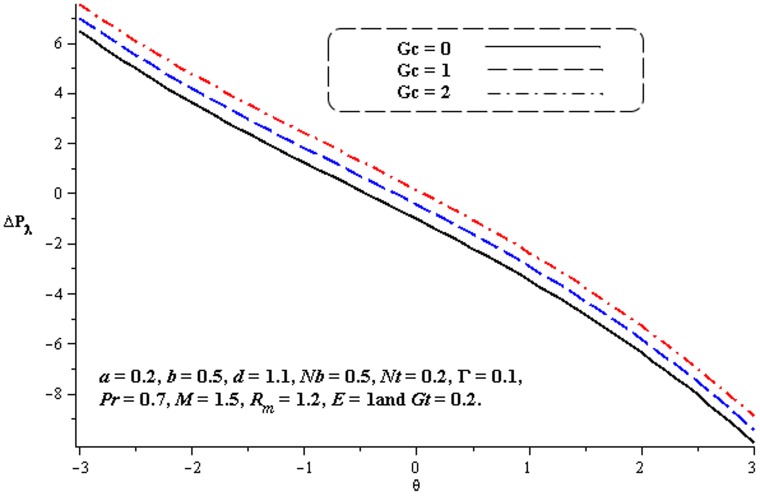
Influence of 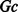 on 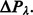

**Figure 6:**
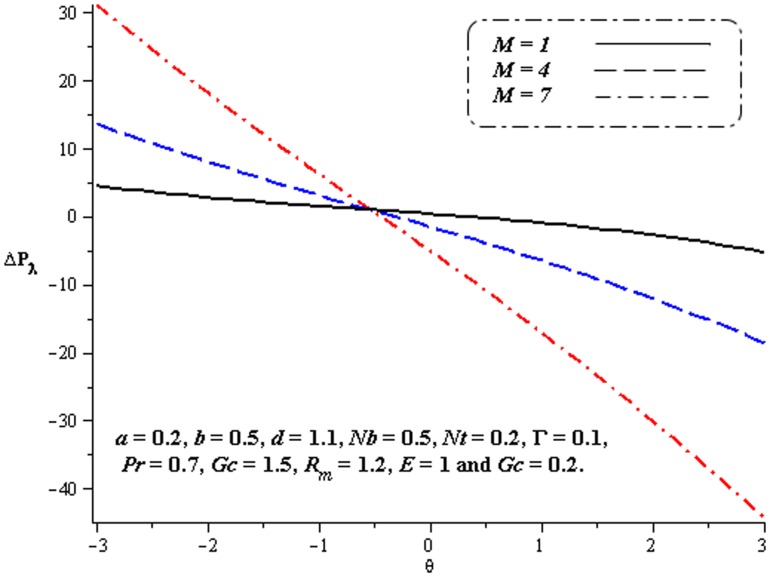
Influence of 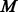 on 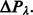

This subsection illustrates the behavior of emerging parameters 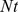, 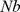, 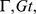 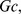 and 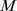 on pressure rise per wavelength 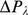. The dimensionless pressure rise per wavelength versus time-averaged flux 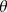 has been plotted in the Figs. 1–6. Here the upper right-hand quadrant 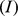 denotes the region of peristalsis pumping, where 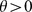 (positive pumping) and 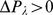 (adverse pressure gradient). Quadrant 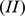, where 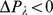 (favorable pressure gradient) and 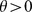 (positive pumping), is designated as augmented flow (copumping region). Quadrant 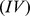, such that 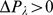 (adverse pressure gradient) and 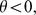 is called retrograde or backward pumping. The flow is opposite to the direction of the peristaltic motion and there is no flow in the last (Quadrant 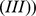. There is an inverse linear relation between 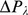 and 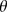. It is noticed from Figs. 1–2 and 4–5 that 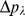 increases with 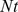, 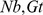 and 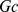 in all the pumping regions. Fig. 3 shows that pumping rate increases by increasing 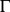 in pumping region. There are specific values of 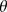 for which there is no difference between viscous and third order nanofluids. On the other hand, in the copumping region the pumping rate decreases with the increase in Deborah number. Fig. 6 shows that 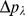 decreases with 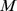 in copumping region.

### 4.2 Flow characteristics

**Figure 7:**
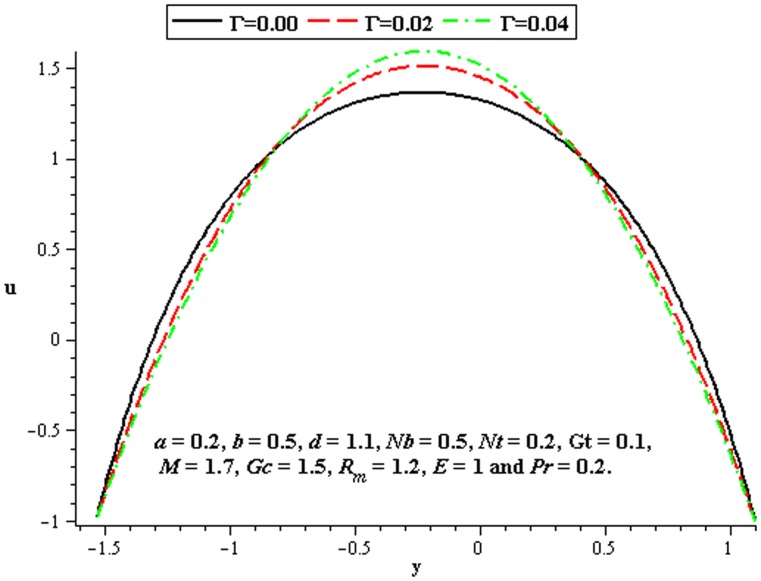
Influence of 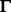 on 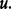

**Figure 8:**
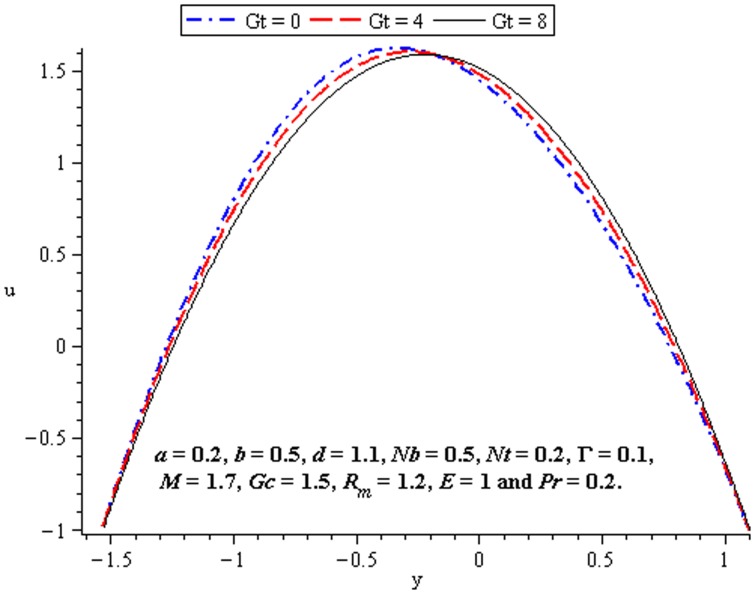
Influence of 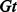 on 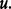

**Figure 9:**
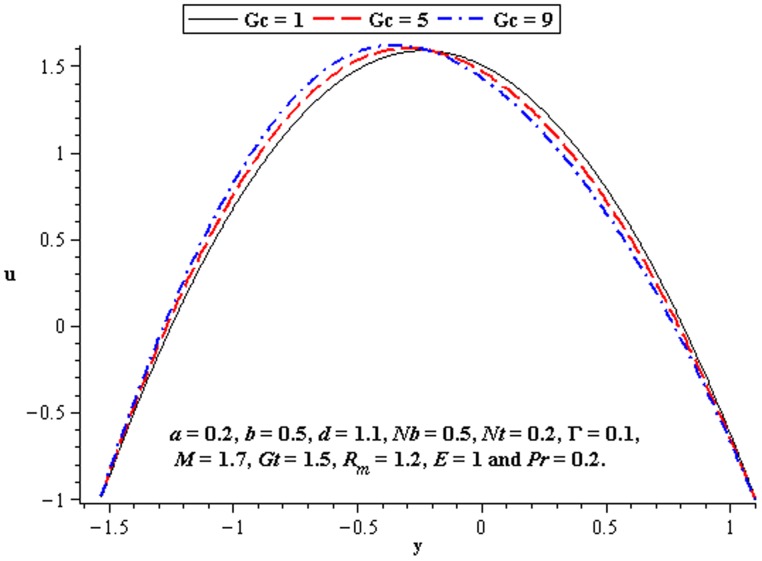
Influence of 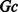 on 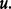

**Figure 10:**
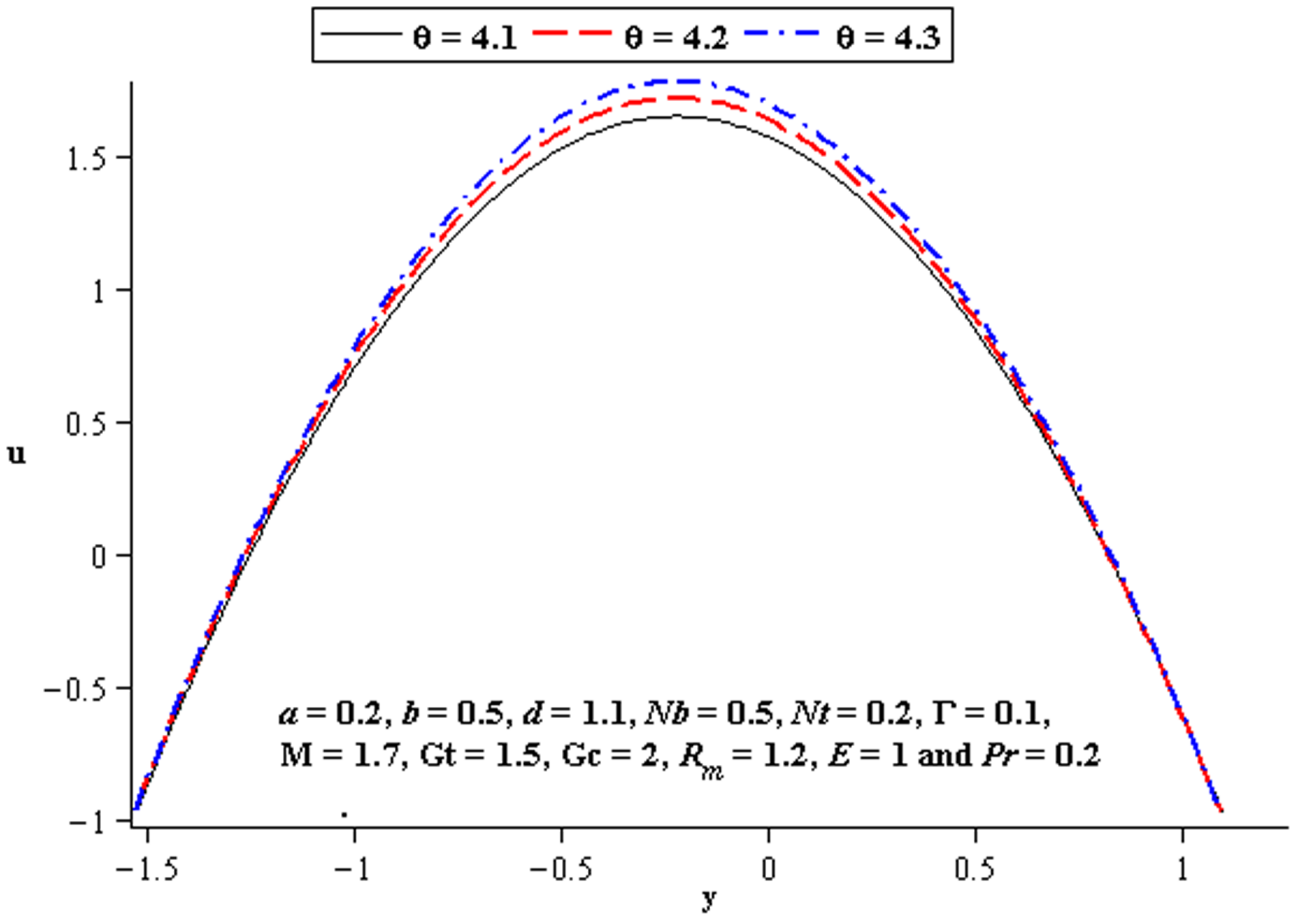
Influence of 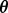 on 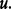

**Figure 11:**
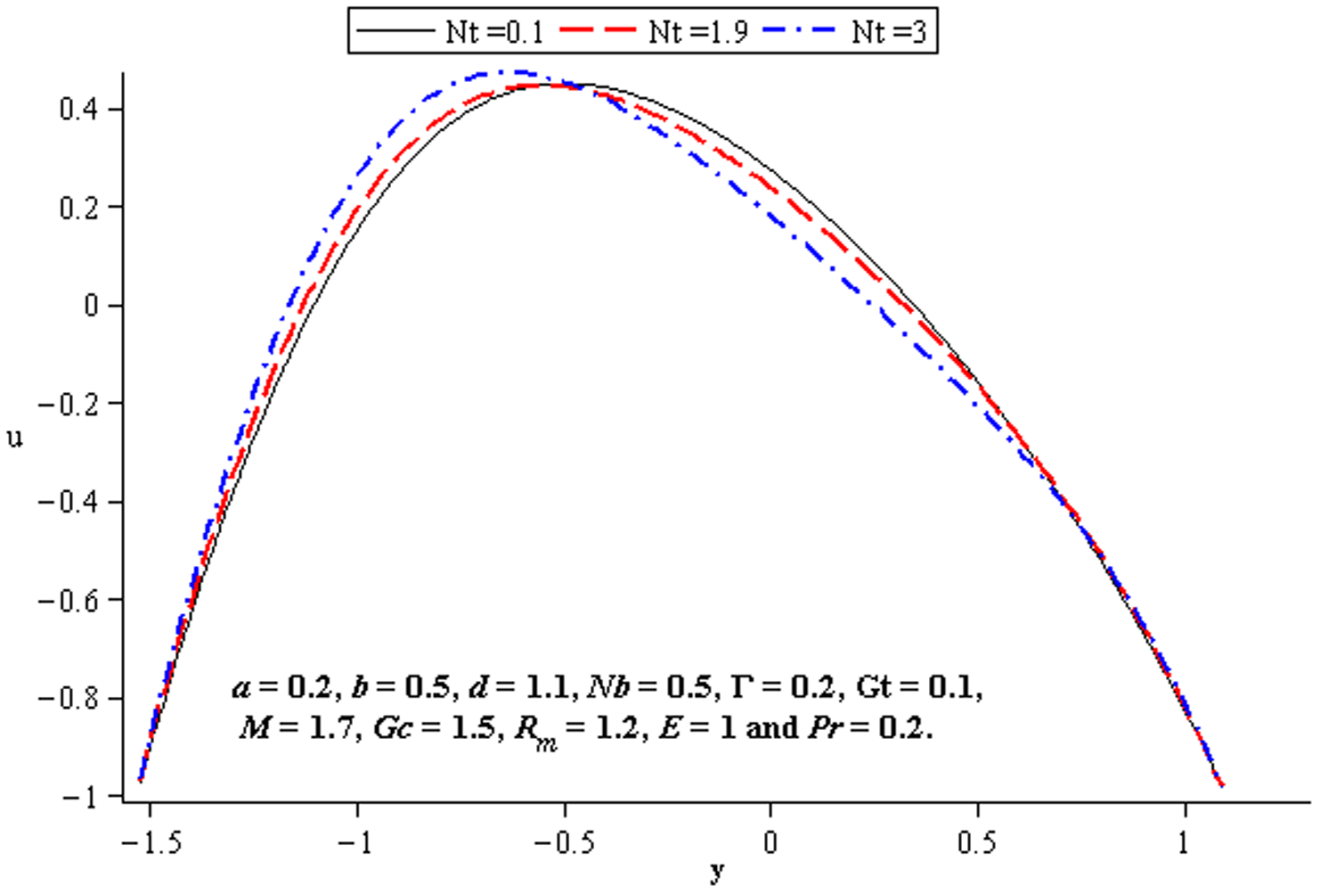
Influence of 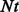 on 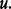

**Figure 12:**
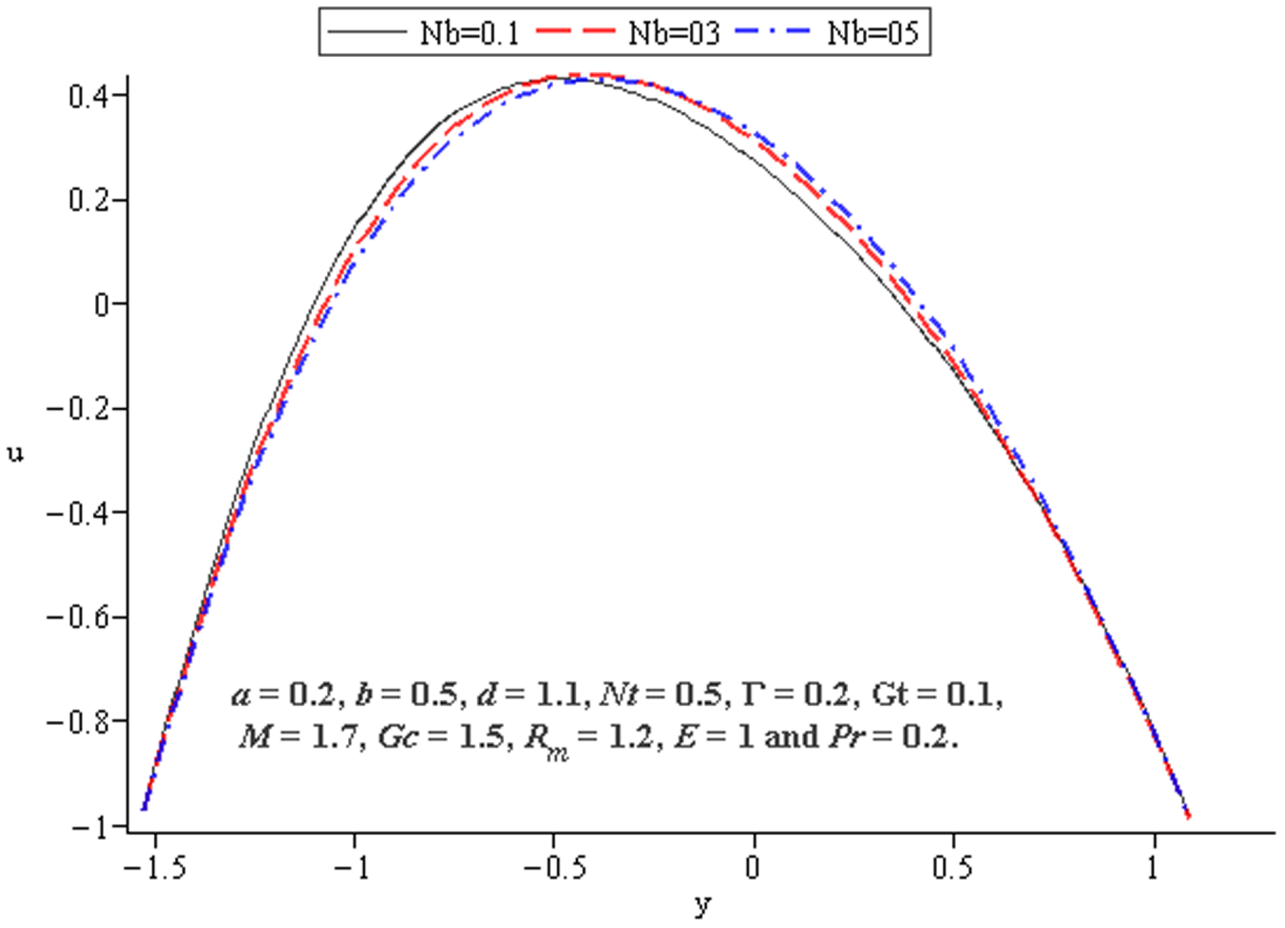
Influence of 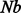 on 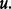

**Figure 13:**
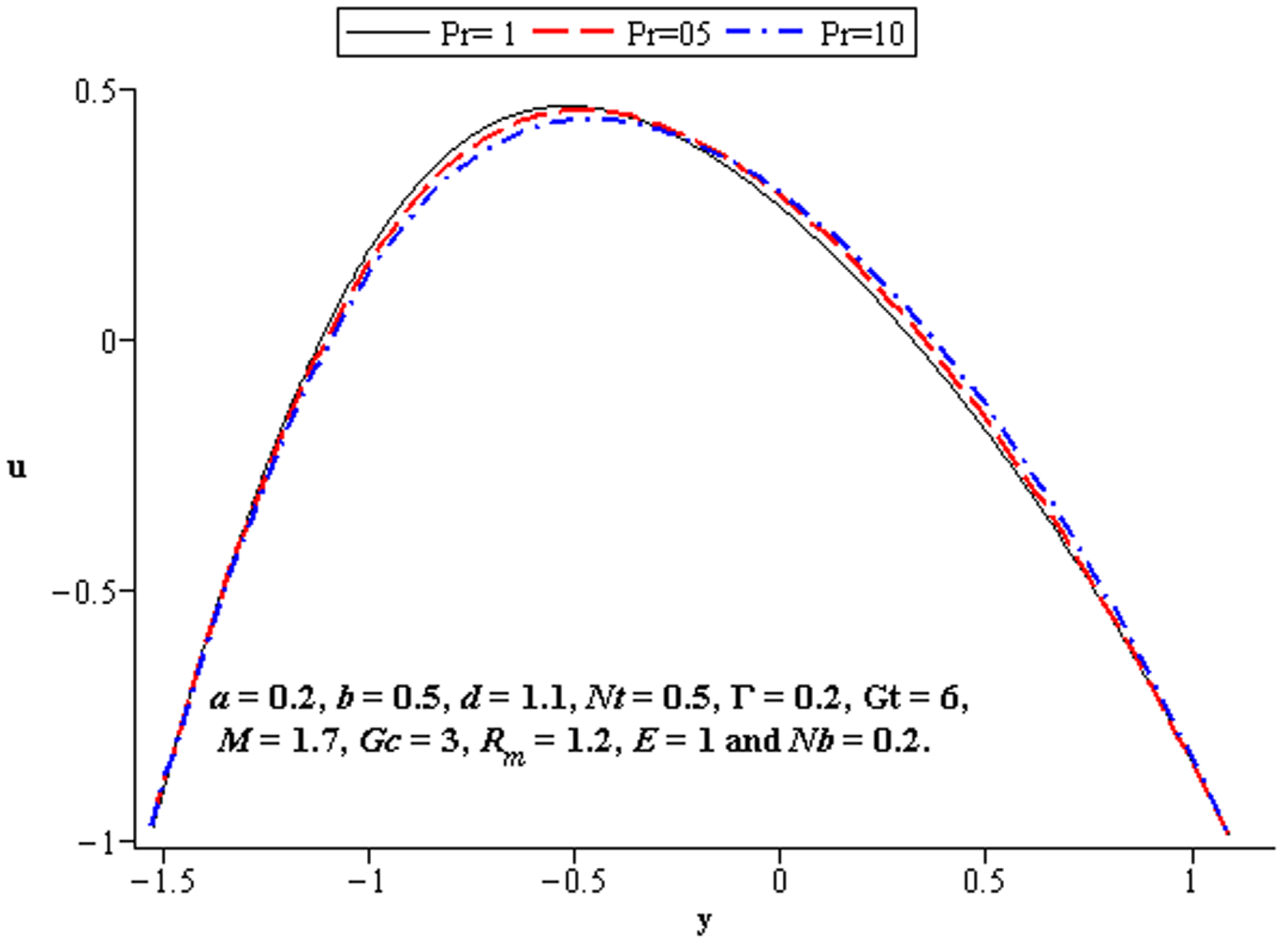
Influence of 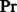 on 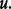

The variations of 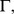 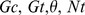, 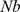, and 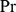 on the velocity have been plotted in this subsection. Fig. 7 shows that there is an increase in velocity at the centre of the channel when 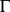 increases. We see a little influence of Deborah number on velocity near the walls of channel. However, magnitude of the velocity of third order nanofluid is more than viscous nanofluid. Figs. 8 and 9 depict the influence of local and mass Grashof number. Clearly the velocity increases near the lower wall. Increase in 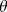 supports the motion in the channel which is shown in Fig. 10. Fig. 11 shows the influence of 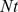 on velocity distribution. Interestingly an increasing thermophoresis leads to an increase in the fluid velocity at the lower wall of channel. There is a considerable variation near the walls 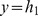 and 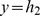 for 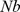 and 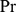 (Figs. 12–13).

### 4.3 Heat transfer characteristics

**Figure 14:**
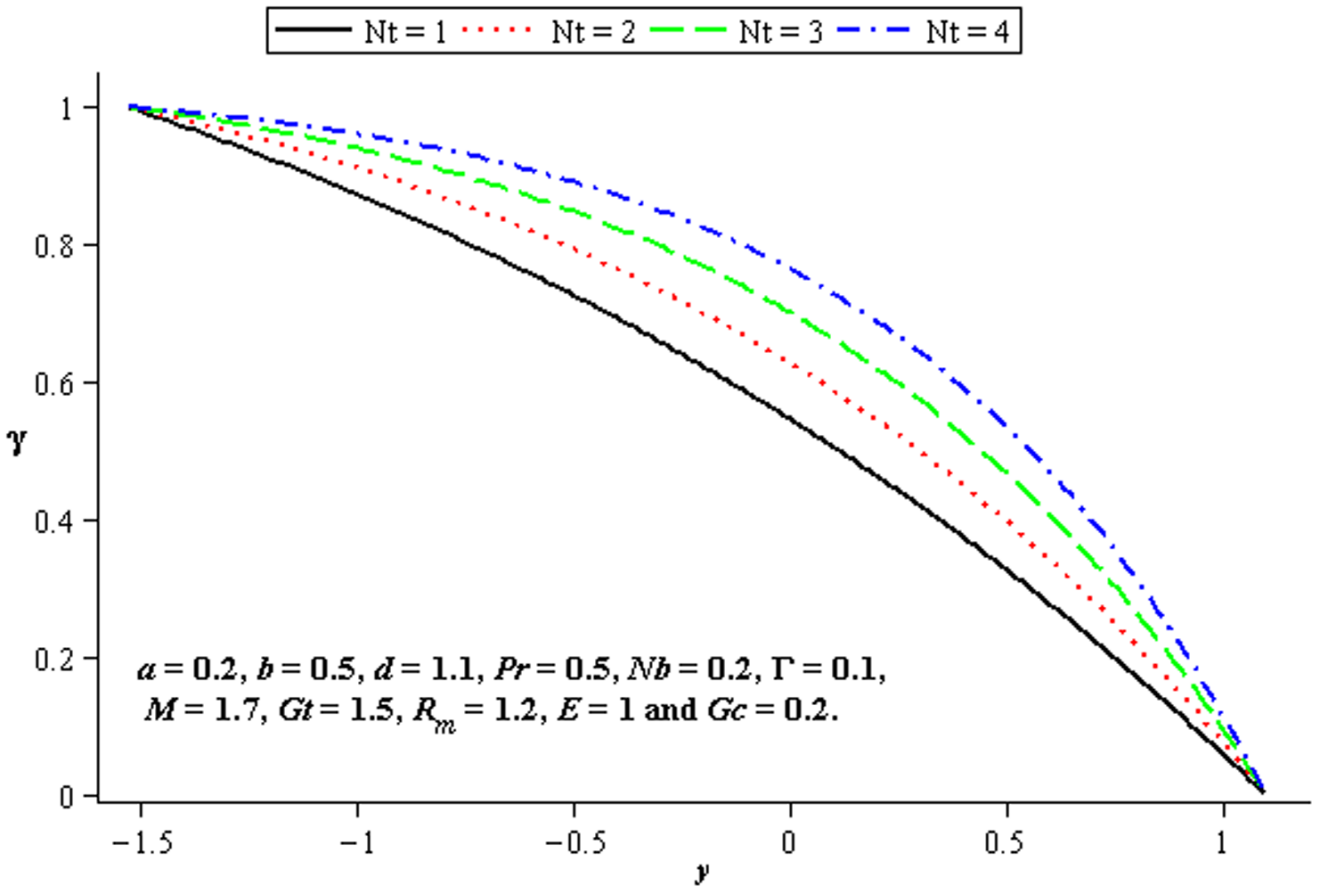
Influence of 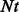 on 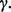

**Figure 15:**
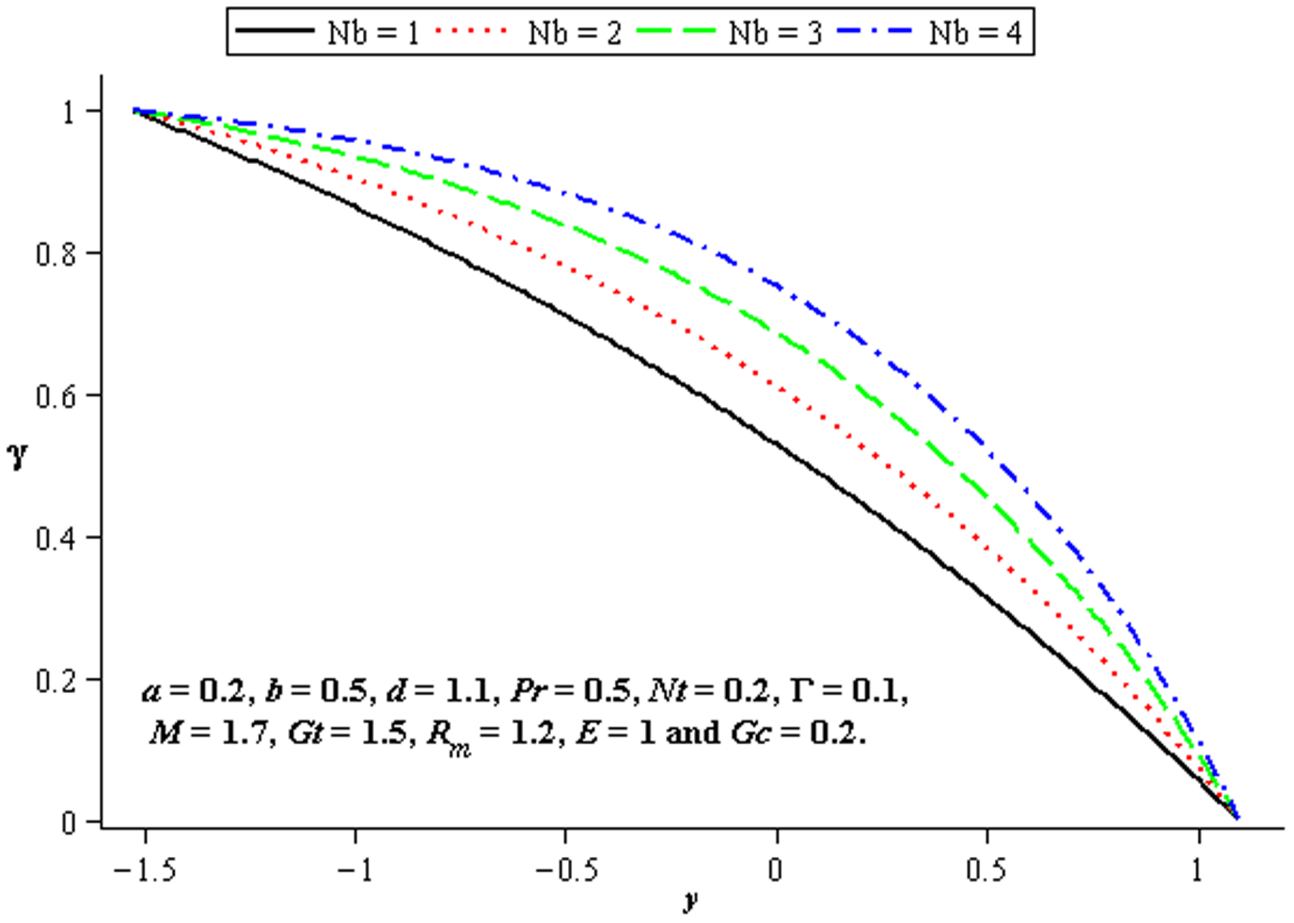
Influence of 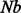 on 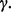

**Figure 16:**
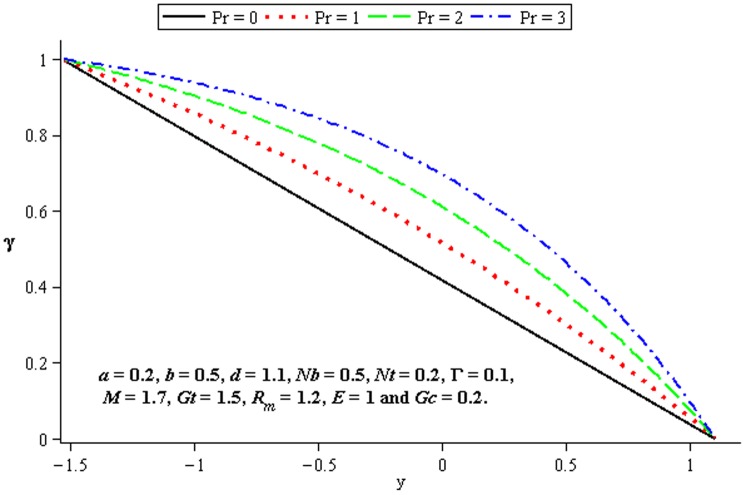
Influence of 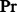 on 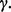

Effect of heat transfer on peristalsis is shown in the Figs. 14–16. Figs. 14 and 15 depict the effects of Brownian motion parameter (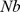) and thermophoresis parameter (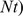 on the temperature profile. One can observe that the temperature profile is an increasing function of 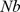 and 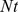 between the walls 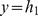 and 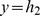. In Fig. 16, we observed the effects of 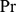 on the temperature profile 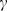 by fixing the other parameters. This Fig. indicates that the temperature increases with the increase of 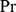.

### 4.4 Mass transfer characteristics

**Figure 17:**
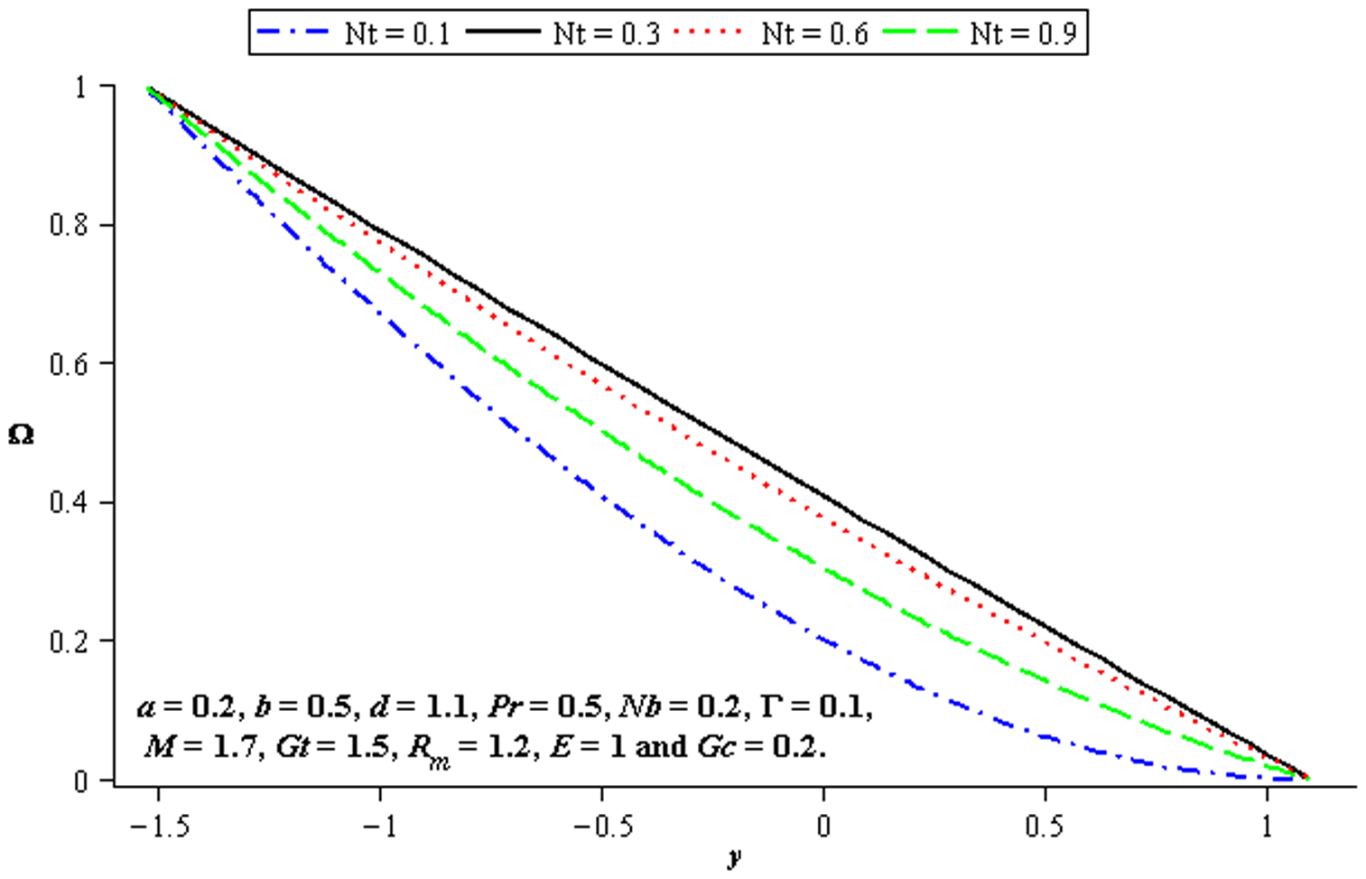
Influence of 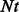 on 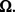

**Figure 18:**
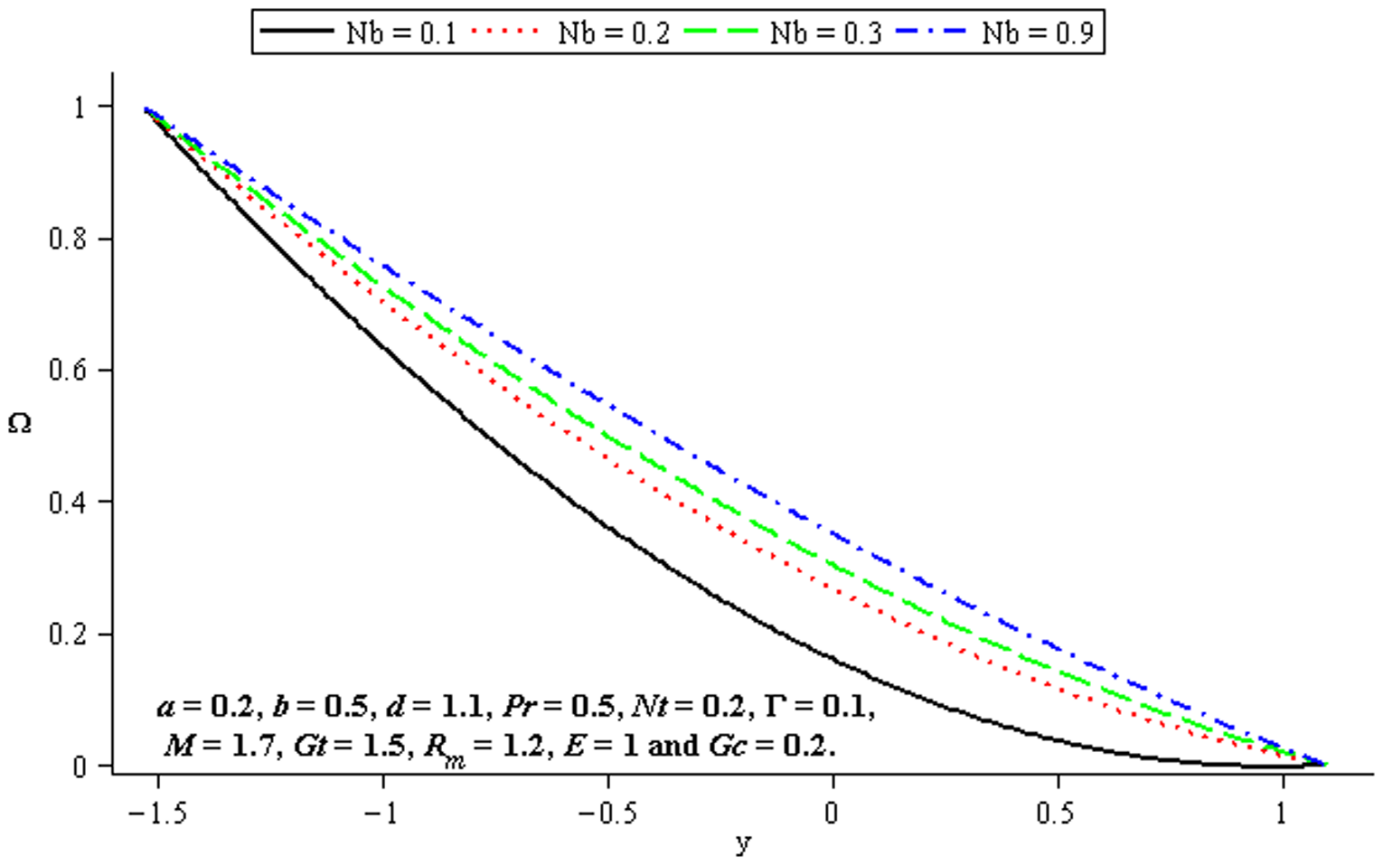
Influence of 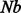 on 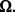

**Figure 19:**
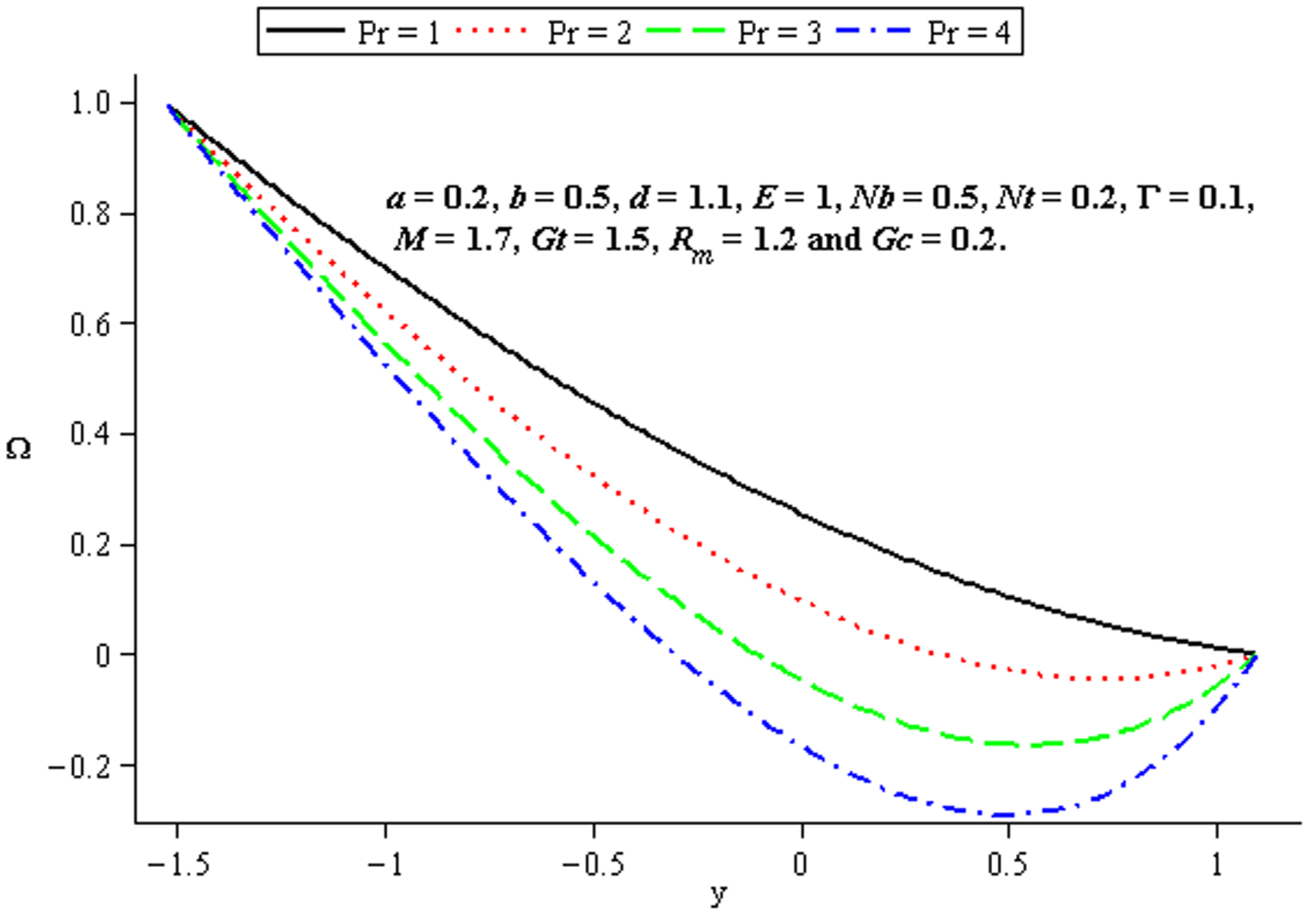
Influence of 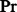 on 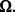

Influence of mass transfer on peristalsis is shown in the Figs. 17–19. Figs. 17 and 18 depict that the concentration distribution increases at the upper and lower walls of channel when 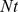 and 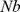 are increased. Fig. 19 shows the effect of 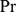 on the concentration when the other parameters are fixed. It shows increasing behavior of 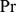 on concentration distribution 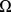 near the walls 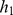 and 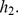


### 4.5 Induced magnetic field characteristics

**Figure 20:**
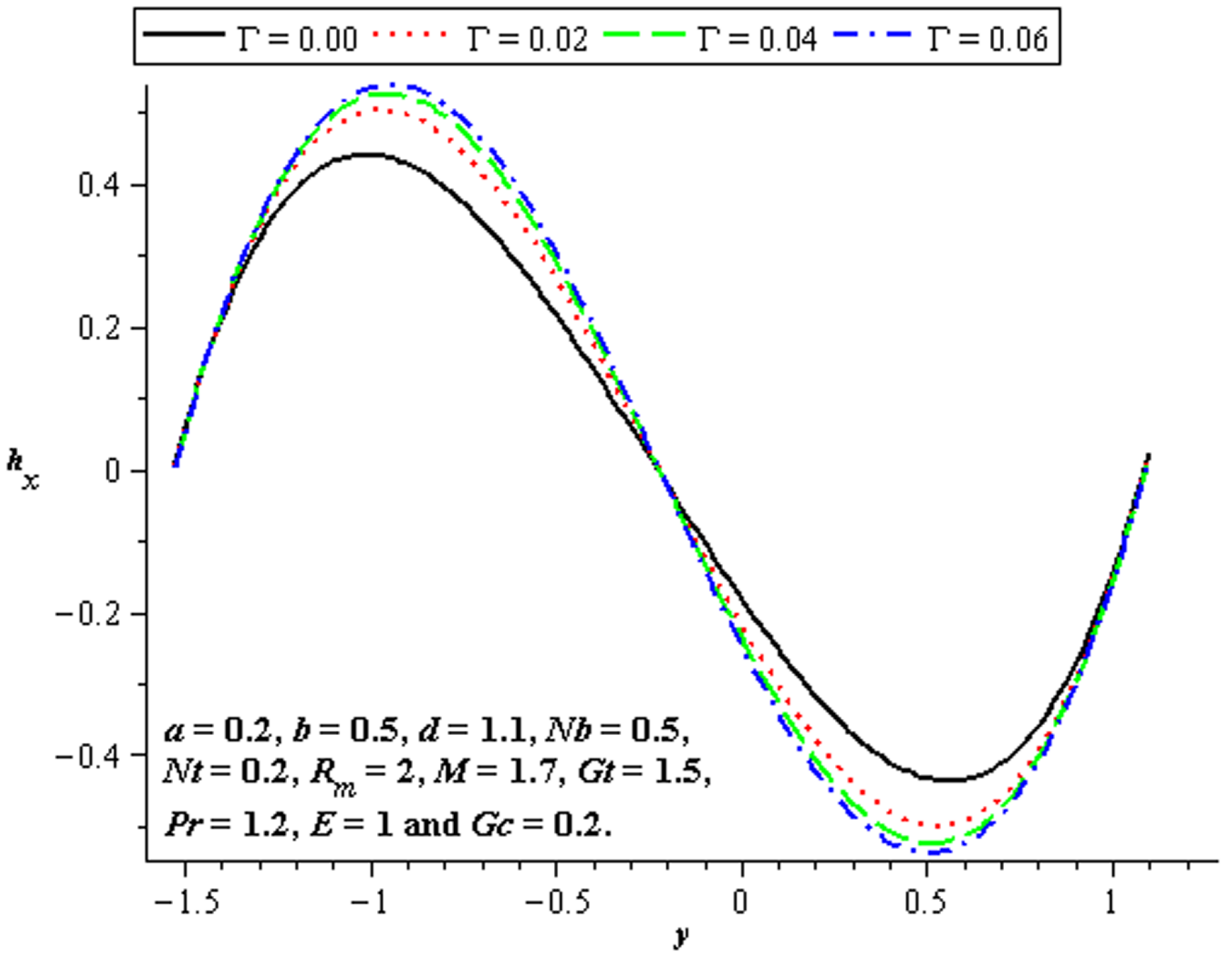
Influence of 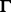 on 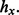

**Figure 21:**
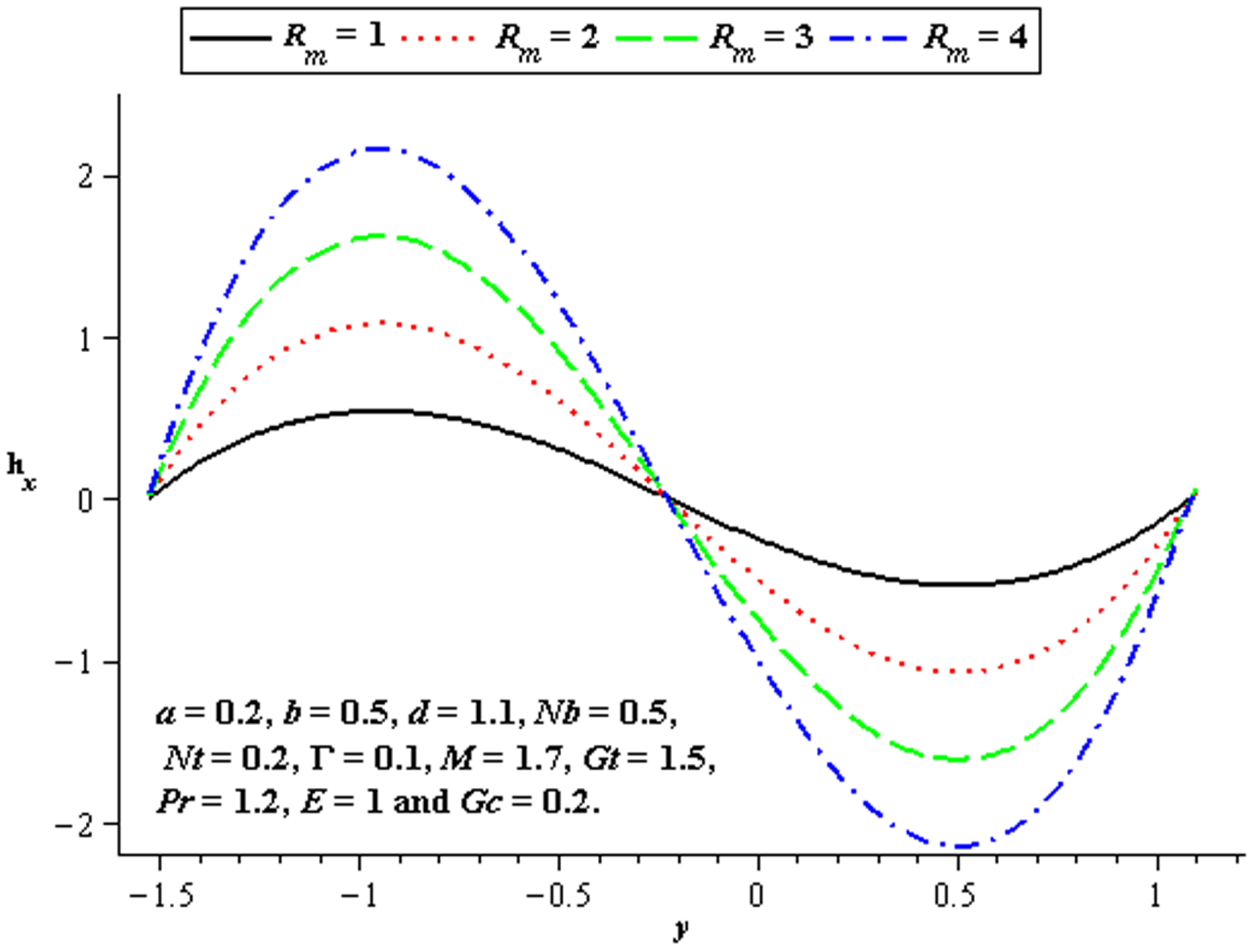
Influence of 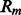 on 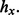

**Figure 22:**
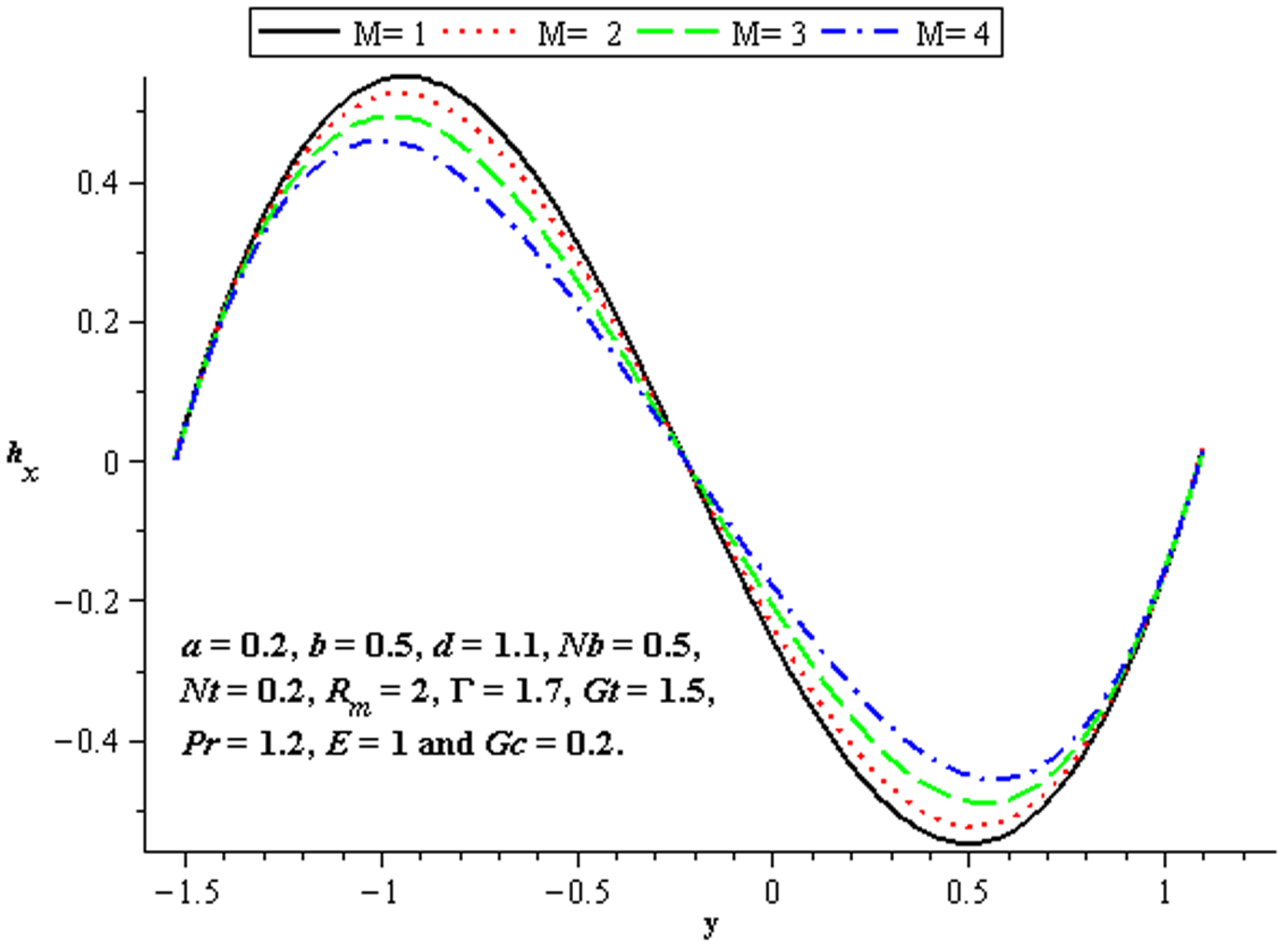
Influence of 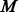 on 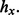

The variations of 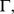 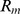 and 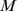 on the induced magnetic field have been plotted in the Figs. 20–22. Fig. 20 shows that there is an increase in 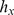 when 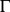 increases. We see that magnitude of the induced magnetic field in third order nanofluid is more than viscous nanofluid. Figs. 21 and 22 depict the influence of 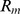 and 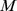. Clearly the 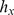 increases near the lower half of channel.

### 4.6 Trapping

**Figure 23:**
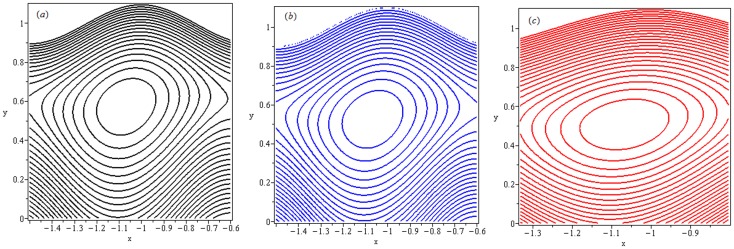
Streamlines for 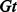 (a): 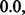 (b): 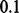 and (c): 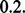

**Figure 24:**
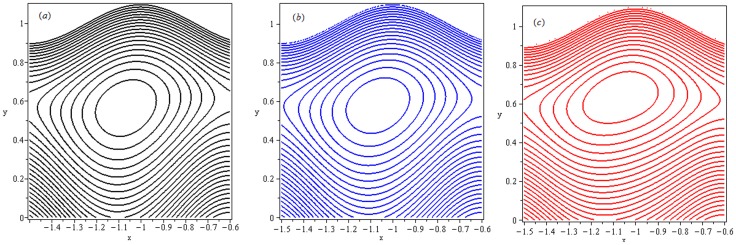
Streamlines for 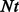 (a): 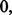 (b): 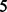 and (c): 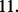

Trapping phenomenon is shown in the Figs. 23 and 24 for different values of 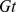 and 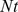 respectively. Trapping is an interesting aspect of peristaltic motion. It is the formation of a bolus of fluid by the closed streamlines. Fig. 23 is made for increasing values of 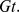 We note that trapping exists for 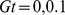, 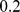 in the upper part of channel. It is observed that number of closed streamlines circulating the bolus reduce in number as we increase the values of local Grashof number. Meanwhile size of trapped bolus increases. Streamlines are plotted in Fig. 24 to see the effects of thermophoresis parameter (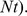 Clearly, the size of trapped bolus increases when 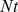 increases from 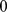 to 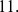 An upper shift and flatness of bolus along with reduced closed streamlines is observed.

## Conclusions

A detailed analysis is presented for peristaltic transport of third order nanofluid in an asymmetric channel with an induced magnetic field and mixed convection. The main findings of the presented study are listed below.

- Pumping rate increases with 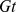, 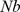 and 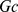 while it decreases with 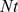 in all pumping regions.
- Velocity distribution is increasing functions of Deborah number at the centre of channel. Absolute value of axial velocity and pressure rise in third order nanofluid is larger than viscous nanofluid.
- Influence of 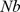 and 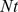 on mass distribution is opposite to temperature distribution.
- Temperature distribution is an increasing function of Brownian motion parameter (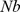) and thermophoresis parameter (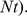

- Induced magnetic field increases with 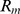 and it decreases with 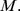
