# Correction: Mixed Convection Peristaltic Flow of Third Order Nanofluid with an Induced Magnetic Field

**DOI:** 10.1371/annotation/c18260c7-d0ff-4f79-82f7-55e9cf254f04

**Published:** 2013-12-20

**Authors:** Saima Noreen

The Funding Statement is incorrect. The Funding Statement should read: "This is to certify that this work is not funded through any external source/research organization including industry etc."

